# Genome-Wide Identification and Expression Analysis of Amino Acid/Auxin Permease (AAAP) Genes in Grapes (*Vitis vinifera* L.) Under Abiotic Stress and During Development

**DOI:** 10.3390/plants14010128

**Published:** 2025-01-04

**Authors:** Xufeng Guo, Na He, Biying Huang, Chongyao Chen, Yanxia Zhang, Xiaoyu Yang, Jie Li, Zhigang Dong

**Affiliations:** 1 College of Horticulture, Pomology Institute, Shanxi Agricultural University, Jinzhong 030800, China; gxfeng2024@163.com (X.G.); hena4776@163.com (N.H.); huangbiying2024@126.com (B.H.); chenchongyao433@163.com (C.C.); zhangyanxia@sxau.edu.cn (Y.Z.); yxy0221@sxau.edu.cn (X.Y.); 2 Grape and Wine Engineering Technology Research Center, Jinzhong 030800, China

**Keywords:** *Vitis vinifera*. L., amino acid transport, collinearity analysis, abiotic stress, development

## Abstract

Amino acids in wine grapes function as precursors for various secondary metabolites and play a vital role in plant growth, development, and stress resistance. The amino acid/auxin permease (*AAAP*) genes encode a large family of transporters; however, the identification and function of the *AAAP* gene family in grapes remain limited. Consequently, we conducted a comprehensive bioinformatics analysis of all *AAAP* genes in grapes, encompassing genome sequence analysis, conserved protein domain identification, chromosomal localization, phylogenetic relationship analysis, and gene expression profiling. This study identified 60 *VvAAAP* genes, distributed on 14 chromosomes and classified into eight subfamilies. Microarray and transcriptome data revealed that most *VvAAAP* genes decrease during development, but *VvAAAP7* and *VvAAAP33* gradually increase. *VvAAAP23* and *VvAAAP46* exhibited significantly higher expression levels, while *VvAAAP30* demonstrated lower expression when subjected to salt and drought stress. *VvAAAP* genes exhibited diverse expression patterns, suggesting that the *AAAP* gene family possesses both diversity and specific functions in grapes. Furthermore, the expression patterns of *VvAAAP* genes analyzed by RT-qPCR facilitate further investigation into the biological functions of individual genes in different tissues. These findings provide valuable insights into the continued analysis of the *AAAP* gene family’s functions in grapes.

## 1. Introduction

Amino acids are essential components in various biological processes across animals, plants, and microorganisms. These processes include protein synthesis, hormone metabolism, neurotransmission, cell growth, energy production, nitrogen metabolism, and urea biosynthesis [1]. The gamma-aminobutyric acid (GABA) transporter, located in the human brain, was the first amino acid transporter identified in nature. Subsequently, the first plant transporter was discovered in a yeast mutant exhibiting defective amino acid transport [2]. In higher plants, amino acids serve as the primary form of organic nitrogen; however, they require the assistance of amino acid transporters (AATs) to traverse the cell membrane [3]. Amino acid transporters comprise a superfamily, with the amino acid/auxin permease (AAAP) gene family representing one of the largest groups within AAT.

Members of the *AAAP* family are present in plants, animals, and microorganisms, with all AAAP proteins sharing a conserved domain (PF01490). Based on sequence similarity and the characteristics of this conserved domain, the family can be classified into eight branches: AAP, lysine and histidine transporter (LHT), gamma-aminobutyric acid transporter (GAT), auxin transporter (AUX), proline transporter (ProT), aromatic and neutral amino acid transporter (ANT), and amino acid-like transporters (ATLa and ATLb) [4]. Members of this family execute diverse functions in plant development and physiological processes, as demonstrated by the roles of AtAAP1, OsAAP8, OsAAP15, and OsAAP4 in amino acid transport, nitrogen metabolism, and plant growth regulation [5,6,7].

Moreover, GATs within the AAAP family specifically transport GABA, which functions as a signaling molecule influencing various plant responses, including carbon/nitrogen balance, pH regulation, nitrogen storage, and defense mechanisms [8,9]. The AAAP family also plays a role in amino acid uptake during seed development. The expression of the high-affinity transporter AAP1 has been detected in the embryo, where it is responsible for the import of amino acids into progeny tissues [10,11]. Furthermore, AAP2 and AAP8 have been identified as contributors to amino acid uptake during seed development [12,13].

The AAP transporter facilitates sugar transport to the syncytium, which subsequently absorbs amino acids as a nitrogen source for biotic stress resistance. Transcriptome analysis has revealed significant upregulation of the AAP amino acid transporter gene in syncytia, and AAP6 mutations affect Arabidopsis growth and amino acid levels, demonstrating the role of amino acid transporters in regulating sieve element composition [14,15]. In Populus tomentosa, *PtAAP11* functions primarily as a proline transporter during xylem cell development [16]. These findings elucidate the diverse biological roles of *AAAP* across various plant species, highlighting its species-specific functional variations.

The *AAAP* gene family plays a crucial role in plant physiology and stress resistance research, demonstrating significant potential value. Research indicates that grapevines accumulate proline to combat stress, and specific amino acids contribute to grape volatile compounds [17,18,19]. During the fermentation process, amino acids serve as the primary nitrogen source for yeast in alcoholic fermentation. Concurrently, amino acids function as precursors of aroma compounds, generating increased levels of alcohols, ester carbon-based compounds, volatile fatty acids, and sulfur compounds [20]. Targeted supplementation of alanine, phenylalanine, and isoleucine during fermentation can enhance fruit ester levels in grape wine [21]. Analysis of the dynamic changes in amino acid and proline consumption by yeast can inform inferences about the aromatic components of Kurchansky and Granatovy grapes and the taste characteristics of future wines [22]. Current research on amino acid transporters in wine grapes is limited, primarily focusing on individual transporter functions or amino acid profiles under specific conditions. There is a lack of comprehensive analysis of the *AAAP* gene family in wine grapes, including sequence similarity, conserved domain distribution, evolutionary relationships, and expression patterns under different growth stages and stress conditions. Utilizing the whole genome database of the wine grape Pinot Noir, this study employs various bioinformatics methods to identify AAAP proteins in wine grapes and to compare their sequence similarity and conserved domain distribution. Using *Arabidopsis (Arabidopsis thaliana)* and *O. sativa (Oryza sativa) AAAP* family members as references, we classified the *AAAP* gene superfamily in grapes, constructed an evolutionary tree, and analyzed their evolutionary characteristics. Moreover, the expression patterns of the *AAAPs* gene in grapes under abiotic stress and during development were analyzed. The reliability of the results was further validated through fluorescence quantitative analysis.

The novelty of this study lies in its comprehensive and integrated approach, combining bioinformatics analysis with experimental validation to provide a deeper understanding of the *AAAP* gene family in wine grapes. By addressing a significant knowledge gap in this field, our study not only contributes to existing research but also paves the way for future investigations, such as identifying functional genes associated with stress resistance and aroma formation in wine grapes. This research is essential for enhancing our comprehension of the amino acid transport mechanisms in wine grapes and advancing the breeding of cultivars with improved stress tolerance and wine quality.

## 2. Results

### 2.1. Identification and Characterization of VvAAAP Genes

The GRAPEDIA portal (https://grapedia.org/, accessed on 30 May 2023) was utilized to obtain the “Pinot Noir” grape PN_T2T genome data and extract the coding sequence (CDS). Relevant data from the grape protein dataset were extracted using the AAAP hidden Markov model (PF01490) (http://pfam.xfam.org, accessed on 20 August 2023). Concurrently, the *Arabidopsis thaliana* AtAAAP sequence was searched using BLASTp (https://blast.ncbi.nlm.nih.gov/Blast.cgi, accessed on 20 August 2023). Both results were submitted to NCBI CDD (http://www.ncbi.nlm.nih.gov/cdd/, accessed on 20 August 2023) to verify the presence of a conserved domain in the obtained sequences, resulting in the identification of 60 genes in the VvAAAP family. ExPASy (https://www.expasy.org, accessed on 21 August 2023), SignalP-4.1 (https://services.healthtech.dtu.dk/services/SignalP-4.1/, accessed on 21 August 2023), and WoLF PSORT (https://wolfpsort.hgc.jp/, accessed on 21 August 2023) were employed to assess and predict characteristics of the members. Sixty *VvAAAPs* were identified from the Pinot Noir genome. The *VvAAAPs* family genes were named *VvAAAP1*–*VvAAAP60* based on their chromosomal location. The amino acid count ranges from 329 (VvAAAP30) to 811 (VvAAAP54), with an average length of 468 amino acids. The molecular weight (MW) of *VvAAAPs* varies between 36,117.8 Da and 89,337 Da, with an average MW of 51,497 Da. The isoelectric point (pI) spans from 5.42 to 9.57, with *VvAAAP27* exhibiting the lowest value and *VvAAAP22* the highest. All grand average of hydropathicity (GRAVY) values are positive, ranging from 0.348 to 0.916. Exon counts vary from 1 to 11, while intron counts range from 0 to 14. The instability index spans from 25.6 to 57.01, with *VvAAAP35* being the least stable and *VvAAAP34* the most stable. The aliphatic index ranges from 95.68 to 128.65, with *VvAAAP15* being the lowest and *VvAAAP29* the highest. In silico subcellular localization prediction of VvAAAP indicates that most VvAAAP proteins are localized in the plasma membrane and vacuole membrane. No signal peptide was predicted in the protein sequence (Table 1).

### 2.2. Analysis of Evolutionary Relationships of the AAAP Gene Family in Different Species

**Figure 1:**
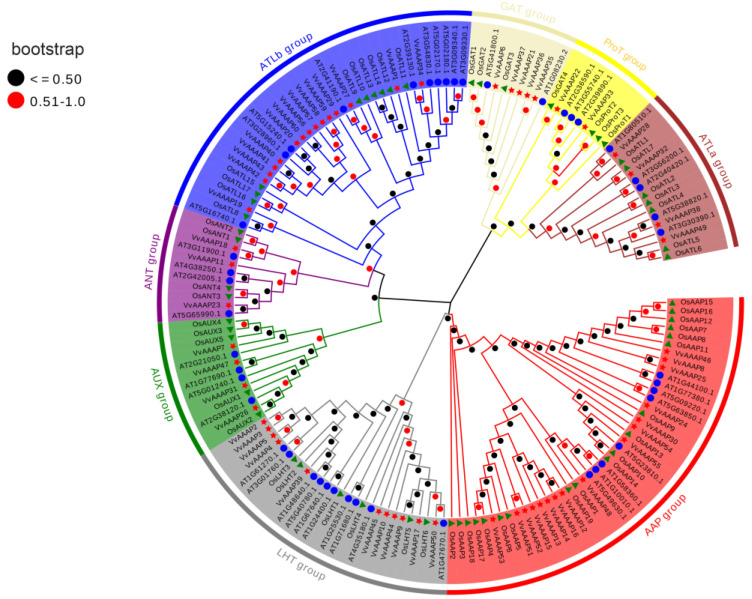
Phylogenetic analysis of the VvAAAP gene family. The neighbor-joining method was employed to align the amino acid sequences of *Arabidopsis thaliana* (blue circle), *Oryza sativa* (green triangle), and *Vitis vinifera*. L. (yellow pentagram) and to conduct a phylogenetic analysis of the AAAPs. The resulting tree displays eight distinct subfamily members, each represented by a different color.

The genome data of rice were retrieved from the Ensembl Plants database (https://plants.ensembl.org/Oryza_sativa/Info/Index, accessed on 21 August 2023), and the CDS and amino acid sequences were extracted. MEGA 11 was utilized to construct a phylogenetic tree, and Evolview (http://www.evolgenius.info/evolview/#login, accessed on 3 September 2023) was employed to enhance the visualization of the evolutionary tree. To examine the evolutionary relationships of the VvAAAP gene family, a phylogenetic tree was constructed using the AAAP protein sequences from *Arabidopsis*, *O. sativa*, and *Vitis vinifera*. Each subfamily comprises AAAP members from these species. The analysis identified 60 members in *Vitis vinifera*, 46 in *Arabidopsis*, and 58 in *O. sativa*. The phylogenetic tree revealed that the 164 AAAPs can be categorized into eight distinct groups. The AAP group contains the highest number of members (43), followed by the ATLb group (35). Conversely, the ProT group is the smallest, comprising only nine members (Figure 1).

### 2.3. Structure, Conserved Motifs, Sequence Alignment, and Phylogenetic Tree Construction of VvAAAPs Family Genes

**Figure 2:**
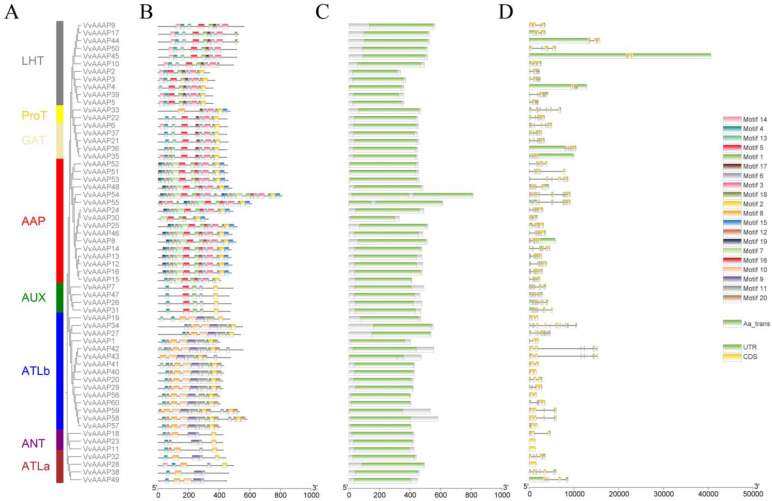
The structure and phylogenetic analysis of the *VvAAAP* gene. (**A**) The phylogenetic tree was constructed using the maximum likelihood method. (**B**) Distribution of motifs in VvAAAP protein, encompassing a total of 20 motifs. (**C**) VvAAAP gene domain, with the Aa_trans domain highlighted in green. (**D**) Gene structure of *VvAAAP*, depicting the untranslated region (UTR) in green and the coding sequence region (CDS) in yellow.

A phylogenetic tree was constructed by comparing the amino acid sequences of VvAAAPs using MAGE 11. The MEME Suite (https://meme-suite.org/meme/tools/meme, accessed on 21 August 2023) was utilized to identify conserved motifs within the grape VvAAAP proteins. The NCBI database (https://www.ncbi.nlm.nih.gov/Structure/bwrpsb/bwrpsb.cgi, accessed on 21 August 2023) was consulted to analyze conserved domains among family members. The phylogenetic analysis revealed that the 60 VvAAAPs can be classified into eight groups: LHT, ProT, GAT, AAP, AUX, ATLb, ANT, and ATLa. The AAP group comprises the largest number of members (16), followed closely by the ATLb group (15), while the ProT group has the fewest members, with only 2 (Figure 2A). Analysis of protein motifs and structural features contributes to our understanding of gene family evolution and the conservation of family members. Gene structure analysis identified a total of 20 conserved motifs. Most VvAAAP proteins contain motifs 14, 4, 13, 5, 1, 17, 6, 3, 18, and 2, whereas motifs 20 and 11 are the least common, being found only in the ATLb subfamily. The ANT and ATLa subfamilies exhibit the smallest number of motifs (Figure 2B). Each VvAAAP protein includes an Aa_trans domain, with VvAAAP54, VvAAAP55, VvAAAP42, and VvAAAP43 possessing two Aa_trans domains (Figure 2C). The AAP and AUX groups exhibit the highest abundance of exons and introns, with *VvAAAP54* having a maximum of 15 exons and 14 introns. Notably, *VvAAAP11*, *VvAAAP23*, and *VvAAAP28* lack introns. Generally, most exons range from 3 to 8, while most introns range from 2 to 7 (Figure 2D).

### 2.4. Chromosome Localization, Collinearity, and Duplication Analysis of VvAAAPs

**Figure 3:**
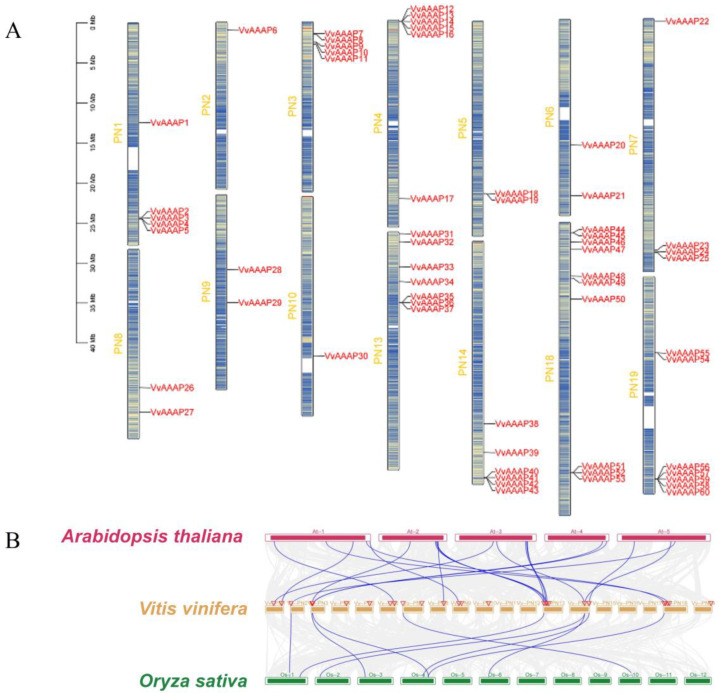
Chromosomal distribution and collinearity analysis of the *VvAAAPs*. (**A**) *VvAAAPs* are depicted on chromosomes; the scale bar on the left indicates chromosome length in Mb. (**B**) Collinearity relationships of *AAAP* genes among *Vitis vinifera*, *Arabidopsis thaliana*, and *Oryza sativa*. Identified collinear genes are connected by blue lines.

**Figure 4:**
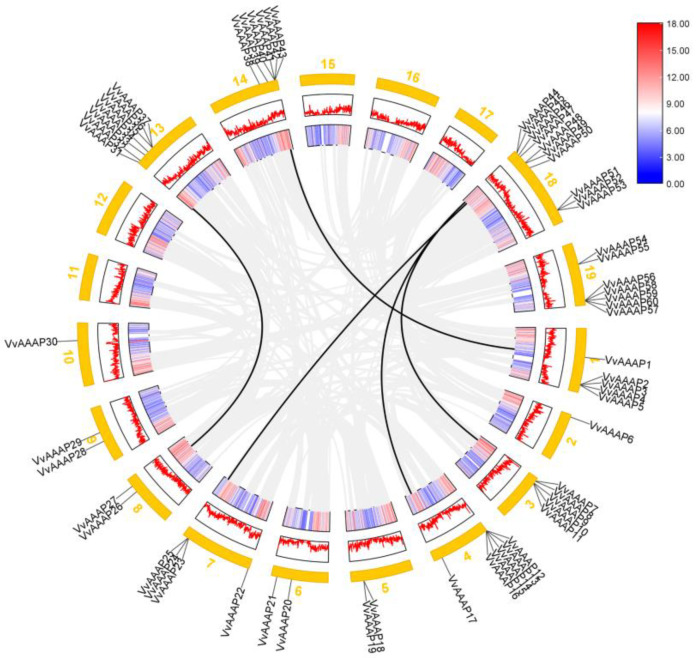
Duplication analysis of the *VvAAAP* genes within grapes. The gray lines in the background indicate all collinear blocks and the black lines indicate segmental duplication pairs of *VvAAAP* genes in grapes.

Chromosome localization analysis reveals that members of the *VvAAAP* family are distributed across various chromosomes, with the exception of chromosomes 11, 12, 15, 16, and 17. Chromosome 18 exhibits the highest concentration of *VvAAAPs*, harboring ten genes, while chromosomes 2 and 10 each contain only one gene (Figure 3A). Collinearity analysis of gene families is crucial for elucidating evolutionary relationships among species; therefore, comparative collinearity analyses were performed between *Vitis vinifera*, *Arabidopsis*, and *O. sativa*. The analysis identified 20 collinear gene pairs between *Arabidopsis* and *Vitis vinifera*, with these *VvAAAPs* distributed across nine chromosomes (Chr1, 2, 3, 6, 8, 9, 13, 14, and 18), including six pairs on chromosome 13. Furthermore, nine collinear gene pairs were detected between *O. sativa* and *Vitis vinifera*, with these *VvAAAPs* located on six chromosomes (Chr2, 3, 7, 13, 14, and 18), of which three pairs are situated on chromosome 14. The results indicate a closer collinear relationship between *Vitis vinifera* and *Arabidopsis* compared to that between *O. sativa* and *Vitis vinifera* (Figure 3B). Gene replication is a common phenomenon in the process of plant evolution. Through gene replication, plants can obtain more gene copies, thereby increasing the diversity and quantity of gene families. The analysis identified five pairs of duplicated gene fragments, including *VvAAAP1/40*, *VvAAAP9/45*, *VvAAAP17/44*, *VvAAAP25/46*, and *VvAAAP26/31* (Figure 4).

### 2.5. Identification of Cis-Acting Elements in the Promoter Region of VvAAAPs

**Figure 5:**
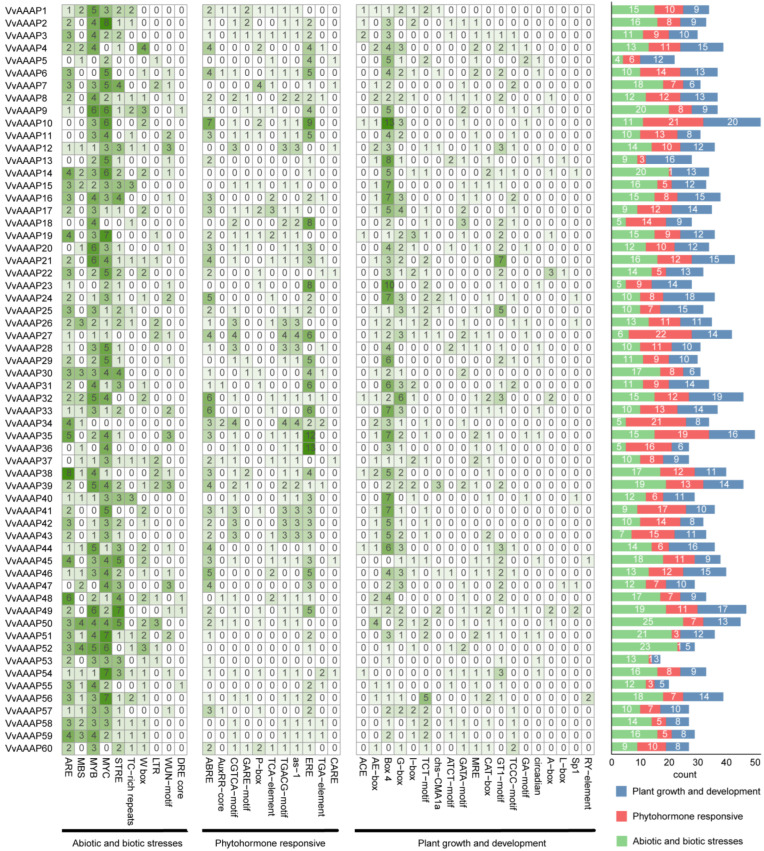
Identification of *cis*-acting elements of *VvAAAP* genes. The *cis*-acting elements are categorized into three groups based on their functions: abiotic and biotic stresses, phytohormone responsiveness, and plant growth and development. The left side indicates the function of *cis*-acting elements and their corresponding numbers. The number of each type of *cis*-acting element in each promoter sequence is displayed on the right side using different colored bars. Each row represents a gene, which is identified on the leftmost side.

The 2000 bp sequence upstream of the start codon of *VvAAAPs* was analyzed using PlantCARE (http://bioinformatics.psb.ugent.be/webtools/plantcare/html, accessed on 1 November 2023) to predict *cis*-regulatory elements within the promoter region. The *cis*-acting elements of *VvAAAPs* were classified into categories related to abiotic and biotic stresses, phytohormone responsiveness, and plant growth and development. Abiotic and biotic stress-related elements were the most numerous, totaling 782, followed by plant growth and development with 673, and phytohormone responsiveness with 579. The *cis*-elements associated with abiotic and biotic stresses were predominant in most gene promoter sequences. Among the 10 selected abiotic and biotic stress *cis*-elements, MYB-related elements (MBS and MYB) and MYC were most abundant in the promoter regions of *VvAAAPs*, with *VvAAAP50* containing the highest number. Regarding phytohormone responsiveness, abscisic acid response elements (ABRE) were most prevalent, identified in 38 genes, with *VvAAAP10* containing the highest number at seven elements. Among the *cis*-elements related to plant growth and development, those associated with light responsiveness (ACE, G-box, box 4, MRE, and ATCT motif) were the most abundant and present in all genes (Figure 5).

### 2.6. Expression Analysis of VvAAAP in Different Wine Grape Varieties and Development Stages

**Figure 6:**
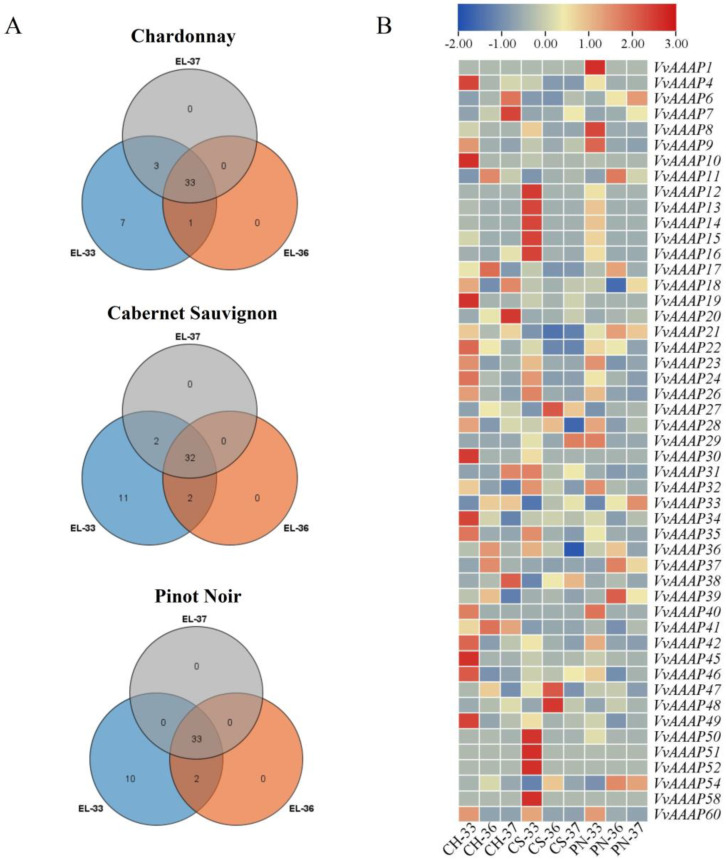
Expression patterns of *VvAAAPs* genes across various wine grape cultivars and developmental stages. (**A**) The quantity of differentially expressed *VvAAAPs* genes at three developmental stages in distinct grape varieties. (**B**) Heat map illustrating the expression patterns of *VvAAAPs* genes during fruit development in different wine grape varieties. The color gradient in the figure represents expression levels, with blue indicating low expression and red denoting high expression. All data have undergone transformation to enhance contrast. CH: Chardonnay, CS: Cabernet Sauvignon, PN: Pinot Noir. EL-33: berries still hard and green, EL-36: berries with intermediate Brix values, EL-37: berries not quite ripe.

Through transcriptome sequencing analysis, we examined the *VvAAAP* gene expression during grape development in Chardonnay, Cabernet Sauvignon, and Pinot Noir varieties, focusing on three developmental stages. Approximately 33 genes are expressed across all three varieties at different stages. Notably, EL-36 and EL-37 stages lack unique expressed genes. Moreover, the EL-33 stage exhibits the highest number of characteristically expressed genes among the three varieties, with 7, 10, and 11 genes identified, respectively (Figure 6A). The analysis revealed that a total of 49 *VvAAAP* genes were expressed across all samples. Significantly, 34 genes showed higher expression levels during the EL-33 stage compared to other stages, with 15 genes expressed in Chardonnay, 11 in Cabernet Sauvignon, and 8 in Pinot Noir. Notably, *VvAAAP12*, *VvAAAP13*, *VvAAAP14*, and *VvAAAP15* were exclusively expressed in the EL-33 stage of each variety. Additionally, *VvAAAP1* was uniquely expressed in the EL-33 stage of Pinot Noir, while *VvAAAP52* and *VvAAAP58* were detected only in Cabernet Sauvignon.

Throughout berry development, the majority of genes exhibited a decreasing expression pattern, with 12, 11, and 9 genes demonstrating this trend in Cabernet Sauvignon, Pinot Noir, and Chardonnay berries, respectively. In contrast, the expression levels of *VvAAAP7* and *VvAAAP33* increased gradually during berry development. Conversely, the expression of *VvAAAP22*, *VvAAAP26*, *VvAAAP32*, *VvAAAP35*, and *VvAAAP60* decreased as the berries matured (Figure 6B).

### 2.7. Expression Analysis of VvAAAP in Different Abiotic Stresses

**Figure 7:**
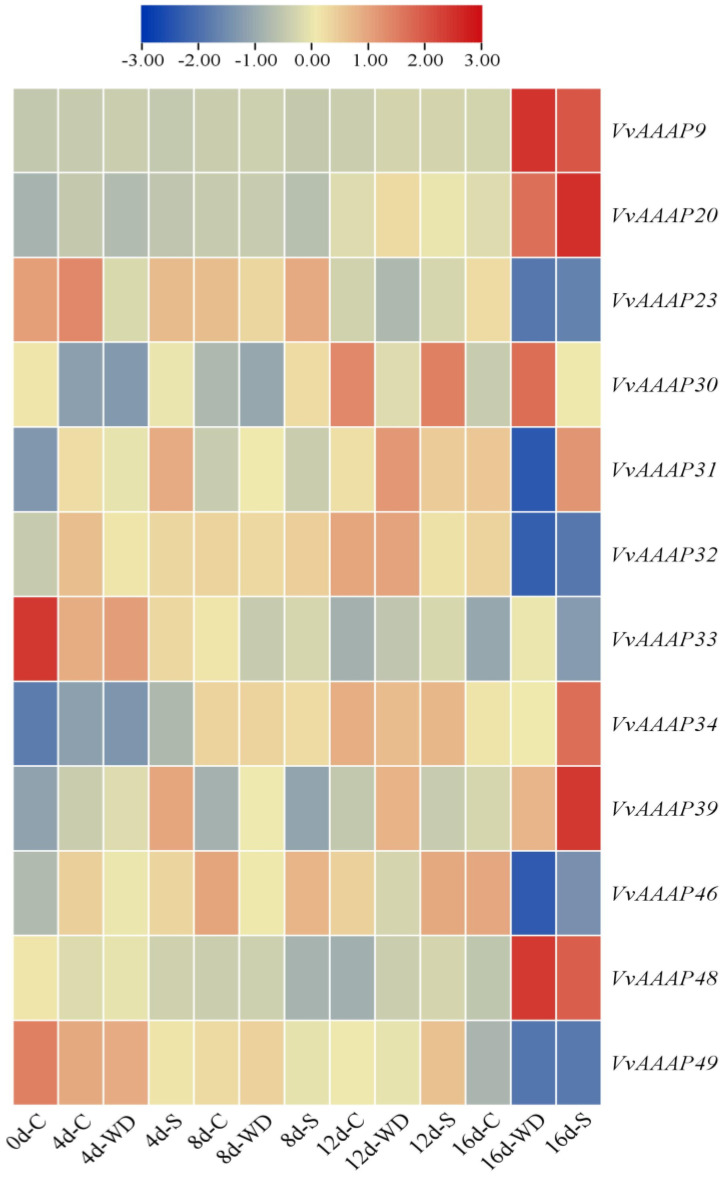
Heat map depicting expression patterns of *VvAAAP* genes in response to water deficit irrigation and salt stress. The color gradient represents expression levels, with blue indicating low expression and red indicating high expression. All data has been transformed to enhance contrast. C: Control, WD: Water deficit irrigation, S: Salt stress.

The expression patterns of *VvAAAP* genes under various abiotic stresses were analyzed using Microarray data (Figure 7). The analysis revealed that 12 *VvAAAP* genes were expressed in the apical tissues of new shoots subjected to salt and drought stress. Notably, *VvAAAP23* and *VvAAAP46* exhibited significantly higher expression levels, while *VvAAAP30* demonstrated lower expression. On the 16th day of each treatment, *VvAAAP9*, *VvAAAP20*, and *VvAAAP48* showed a significant increase in expression, whereas *VvAAAP23*, *VvAAAP32*, *VvAAAP46*, and *VvAAAP49* displayed a significant decrease. As salt stress intensity increased, the expression of *VvAAAP9* and *VvAAAP34* progressively increased, while *VvAAAP32* and *VvAAAP33* gradually decreased. Similarly, under increasing drought stress, *VvAAAP9* and *VvAAAP20* expression also increased, whereas *VvAAAP49* showed a gradual decline. The gene expression trends demonstrated similarities between salt and drought stress conditions.

### 2.8. RT-qPCR Analysis of VvAAAP Genes Across Developmental Stages and Tissues

**Figure 8:**
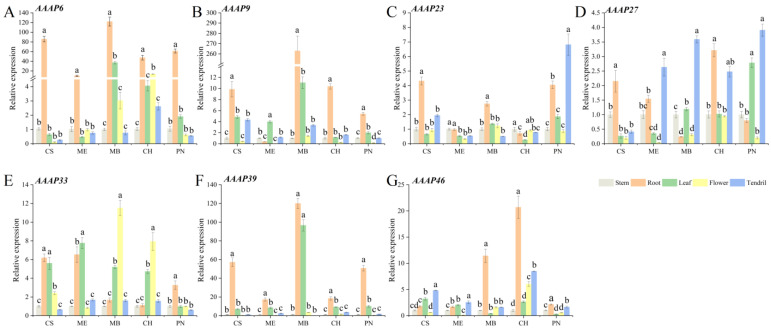
RT-qPCR analysis of *VvAAAP* genes in diverse tissues from multiple wine grape varieties. CS: Cabernet Sauvignon, ME: Merlot, MB: Muscat Blanc, CH: Chardonnay, PN: Pinot Noir. Three biological replicates were performed. Bars graphs and error bars represent average and SE, respectively. Significant differences were indicated by lowercase letters based on Fisher LSD-test between different grape varieties of the same sampling period (*p* ≤ 0.05).

Evaluating gene expression levels across various tissues from different wine grape varieties enhances our understanding of the functional roles of distinct subfamily genes. An analysis of the relative expression levels of seven *VvAAAP* family genes revealed notable patterns. *VvAAAP6* exhibited the highest expression level in the roots across all varieties. Similarly, *VvAAAP9* demonstrated peak expression in roots, with the exception of Merlot. *VvAAAP9* and *VvAAAP39* displayed comparable expression patterns, with maximum levels observed in the roots and leaves of Muscat Blanc. *VvAAAP27* showed elevated expression in tendrils compared to other tissues. In Muscat Blanc and Chardonnay, *VvAAAP33* exhibited higher expression levels in leaves and flowers relative to other tissues, while in Pinot Noir, Cabernet Sauvignon, and Merlot, its expression was elevated in roots and leaves. Additionally, *VvAAAP46* demonstrated peak expression levels in the roots of both Chardonnay and Muscat Blanc (Figure 8).

**Figure 9:**
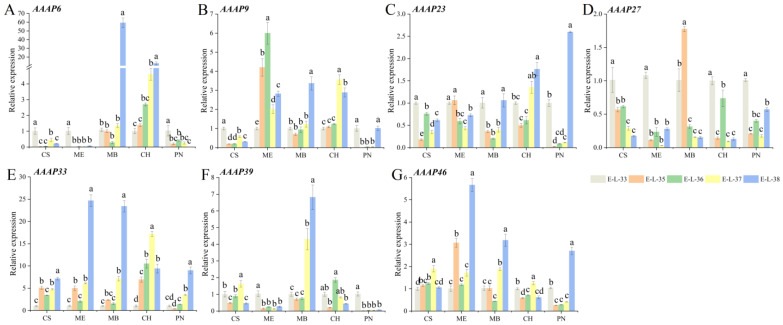
RT-qPCR analysis of *VvAAAP* genes across developmental stages from different wine grape varieties. CS: Cabernet Sauvignon, ME: Merlot, MB: Muscat Blanc, CH: Chardonnay, PN: Pinot Noir. EL-33: Berries still hard and green, EL-35: Berries begin to color and enlarge, EL-36: Berries with intermediate Brix values, EL-37: Berries not quite ripe, EL-38: Berries harvest-ripe. Three biological replicates were performed. Bars graphs and error bars represent average and SE, respectively. Significant differences were indicated by lowercase letters based on Fisher LSD-test between different grape varieties of the same sampling period (*p* ≤ 0.05).

The expression patterns of *VvAAAP6* varied among grape varieties during development. In Muscat Blanc and Chardonnay, *VvAAAP6* expression increased progressively, peaking at the EL-38 stage. Conversely, in Cabernet Sauvignon, Merlot, and Pinot Noir, *VvAAAP6* expression reached its maximum during the EL-33 period. *VvAAAP9* expression also showed a gradual increase in Muscat Blanc and Chardonnay, while in Merlot, it initially increased and subsequently decreased. *VvAAAP23* expression was lowest at the EL-35 and EL-36 stages across all varieties, generally exhibiting a trend of decreasing followed by increasing. *VvAAAP27* demonstrated the highest expression level at the EL-33 stage in all varieties except Muscat Blanc. *VvAAAP33* expression peaked at EL-38, with Chardonnay reaching its maximum at EL-37. In Chardonnay, *VvAAAP39* expression was highest at the EL-38 stage, while in Pinot Noir, expression at EL-33 was significantly higher than at other stages. *VvAAAP46* showed peak expression during the EL-38 period in Merlot, Muscat Blanc, and Pinot Noir (Figure 9).

## 3. Discussion

The *AAAP* family genes have been extensively studied in model plants such as *Arabidopsis* [23], *O. sativa* [6], *Populus* [4], and *Medicago truncatula* [24], elucidating their roles in stress resistance, plant growth, and development. Advancements in sequencing technology have expanded the range of identified plants to include *Solanum lycopersicum* [25], *Maize* [26], *Setaria italica* [27], *Cerasus humilis* [28], and *Fragaria vesca* [29]. Despite the publication of the T2T reference genome for Pinot Noir [30], a comprehensive study of the grape *AAAP* gene family has been lacking. This study presents a thorough identification and characterization analysis of the *VvAAAP* gene family in grapes, successfully identifying 60 *VvAAAP* genes from the genome. Comparatively, the number of *AAAP* family genes in grapes is lower than the 82 found in poplar [4], 65 in millet [27], and 71 in wheat [31], but higher than the 43 in Arabidopsis [23], 58 in rice [6], 45 in strawberry [29], and 55 in moso bamboo [32]. The variation in the number of *VvAAAP* family genes in grapes compared to other plants may be attributed to factors such as genome size, gene number, evolutionary history, gene duplication, ecological environment, and gene adaptability. The amino acid composition, relative molecular weight, isoelectric point, signal peptide, and other physical and chemical properties encoded by these genes in grapes exhibit notable differences, suggesting potential diversity in structure and function among the *VvAAAP* family genes. Furthermore, the positive GRAVY values for these genes indicate a likely hydrophilic nature. The variations in the number of exons and introns also reflect the structural complexity of the *VvAAAP* gene family.

In the phylogenetic analysis, 60 *VvAAAPs* were categorized into eight groups: LHT, ProT, GAT, AAP, AUX, ATLb, ANT, and ATLa. This classification aligns with observations in other plant species [28,32]. The number of subfamilies within a gene family results from the interplay of multiple factors, including the frequency of gene duplication events, adaptability, and environmental pressures, as well as genetic drift and population size. This grouping enhances our understanding of the evolutionary relationships within the *VvAAAP* gene family and provides valuable insights for subsequent functional studies. Notably, the AAP group contains the highest number of *VvAAAP* genes, while the ProT group comprises the fewest, potentially reflecting their distinct roles in grape growth, development, and stress response. The diversity of subfamily members varies significantly among species; for instance, whole-genome analysis of *AAAPs* in moso bamboo reveals that the AAP subfamily has the largest representation [32]. Furthermore, in strawberries, the LHT subfamily has the highest number of *AAAP* genes, followed closely by the ATLb type [29].

Chromosome location analysis revealed that *VvAAAP* family genes are distributed across multiple chromosomes in grapes, with varying distribution numbers among these chromosomes. This distribution pattern may be associated with gene family expansion and alterations in chromosome structure. Through comparative analysis of AAAP protein sequences from *Vitis vinifera*, *Arabidopsis*, and *O. sativa*, this study further investigated the evolutionary relationships of the VvAAAP family. The relatively large number of VvAAAPs in grapes and the notable collinear relationship with AAAP proteins of other species suggest evolutionary conservation. Moreover, the more pronounced collinearity between *Vitis vinifera* and *Arabidopsis* compared to *O. sativa* likely reflects a closer evolutionary relationship between the former two species. The collinearity analysis also elucidated evolutionary connections and distinctions between grapes and other species, providing novel insights into the evolutionary relationships among these species.

The identification and analysis of *cis*-acting elements in the promoters of *VvAAAPs* revealed the presence of various regulatory elements within the upstream regions of these genes. A more detailed examination of *cis*-elements associated with hormone responses and critical secondary metabolic pathways suggests the potential involvement of *VvAAAPs* in grape growth, development, and stress responses. Studies indicate that grapevines accumulate proline as a mechanism to combat stress [17]. Additionally, isoleucine, leucine, phenylalanine, and valine function as precursors for higher alcohols and esters, contributing to the formation of volatile compounds in grapes [18,19].

Amino acid levels in plants play a crucial role in regulating growth and are closely associated with plant development and stress resistance. Auxin, a vital plant hormone for survival, is present in all plants [27]. Several *VvAAAP* genes were expressed in the apical tissues of new shoots subjected to salt and water deficit irrigation, with their expression patterns exhibiting a discernible trend as the stress severity increased. This suggests that *VvAAAP* family genes may be involved in the grapevine’s response mechanisms to salt and water deficit irrigation, thus providing potential genetic resources for breeding stress-resistant grape varieties. Furthermore, *AAAP* has been found to be induced by abiotic stresses in other species. A genome-wide identification study identified 65 *SiAAAP* genes in millet and examined their expression patterns induced by salt–alkali stress [27]. Notably, members of the *PeAAP* subfamily displayed significant variations in expression patterns across three types of abiotic stress. Following treatment with these stresses, the expression of most *PeAAP* genes was up-regulated, indicating that *PeAAP* genes may play a significant role in the abiotic stress response of moso bamboo [32].

Transcriptome analysis revealed that the *VvAAAP* gene family demonstrates diverse expression patterns throughout various stages of grape development and across different varieties. This observation suggests that these genes may play a pivotal role in berry growth, development, and quality formation. Notably, genes exhibiting significantly elevated expression levels during specific developmental stages or in particular varieties may possess more crucial functions.

Through RT-qPCR analysis, we evaluated the expression levels of several *VvAAAP* family genes across different tissues and developmental stages. These findings validate the reliability of the transcriptome data and provide crucial insights for a comprehensive understanding of gene functions across various subfamilies. Notably, *AAAP46* exhibits high expression in the roots of Chardonnay and Muscat Blanc, while *AAAP9* demonstrates elevated expression in the roots of four distinct varieties, indicating their potential roles in grape root system development and function. *AAAP46* is categorized within the AAP subfamily, while *AAAP9* belongs to the LHT subfamily. Previous research has shown that *AtLHT1* displays the highest expression levels in roots [33]. Moreover, *OsAAP6* modifies the distribution of several amino acids during early rice seed development and enhances nutrient absorption by roots [6]. The expression level of *AAAP27* in tendrils surpasses that in other tissues, potentially indicating its specific role in tendril development or function. In *Arabidopsis*, *AtATLb1* encodes a specific transporter for lysine and histidine and is highly expressed in floral tissues [34]. *AAAP27* is part of the ATLb subfamily, and tendrils are homologous to flower organs. *FvAAAP10*, homologous to *AtATLb1*, exhibits high expression in all floral structures [29]. Similar patterns were observed in peppers, with several genes showing relatively high expression in specific organs, such as *CaLHT3*, *CaLHT5*, *CaLHT8*, *VAAT1*, and *VAAT6* in flowers; *CaATL4* in fruits; *CaLHT9* and *CaGAT2* in roots; and *CaLHT12* in roots, stems, and leaves [35].

## 4. Materials and Methods

### 4.1. Plant Materials

The experiment was conducted at the vineyard of the Fruit Research Institute of Shanxi Agricultural University, located in Jinzhong City, Shanxi Province, China (38°37′ N, 106°01′ E). This region is situated in the mid-latitude inland, characterized by a warm temperate continental semi-arid monsoon climate, with loess as the predominant soil type. The grape varieties used as test materials included Cabernet Sauvignon, Pinot Noir, Merlot, Chardonnay, and Muscat Blanc, all sourced from self-rooted seedlings. In the experimental field, the spacing between rows was set at 1.0 m × 3.0 m. The shaping utilized a single-arm horizontal trunk, with annual branches pointing vertically upward. A consistent integrated water and fertilizer management technology was employed to regulate water and nutrient supply, with scientifically controlled loads. For each grape variety, 30 plants exhibiting uniform growth and free from pests and diseases were selected, resulting in three biological replicates and a total of 90 plants. Samples for transcriptome sequencing analysis were collected from Cabernet Sauvignon, Pinot Noir, and Chardonnay at EL-33, EL-35, and EL-37 stages. Additionally, samples from Cabernet Sauvignon, Pinot Noir, Merlot, Chardonnay, and Muscat Blanc were collected at EL-33 (berries still hard and green), EL-35 (berries begin to color and enlarge), EL-36 (berries with intermediate Brix values), EL-37 (berries not quite ripe), and EL-38 (berries harvest-ripe) for developmental stage analysis, while roots, stems, leaves, flowers, and tendrils were utilized for RT-qPCR analysis [36,37].

### 4.2. Identification and Characterization of Grape VvAAAP Family Genes

The Pinot Noir grape PN_T2T genome was obtained from the GRAPEDIA portal (https://grapedia.org/, accessed on 30 May 2023) [30]. TBtools v2.096 was used to extract the CDS of all genes from the genomic data and subsequently translate them into amino acid sequences. The gene IDs for the Arabidopsis *AtAAAP* family were obtained from published articles [4], with corresponding amino acid sequences downloaded from TAIR (http://arabidopsis.org, accessed on 20 August 2023) [38]. The Pfam-A.hmm folder was acquired from Pfam (http://pfam.xfam.org, accessed on 20 August 2023). The Simple HMM Search plug-in in TBtools, utilizing the AAAP hidden Markov model (PF01490), was employed to extract relevant data from the grape protein dataset [39]. This process initially identified a collection of AAAP family members. Additionally, a BLASTp (https://blast.ncbi.nlm.nih.gov/Blast.cgi, accessed on 20 August 2023) search was conducted using the *Arabidopsis thaliana* AtAAAP sequence as the query against the protein database to identify VvAAAPs. The results from both methods were submitted to NCBI CDD (http://www.ncbi.nlm.nih.gov/cdd/, accessed on 20 August 2023) to verify the presence of conserved domains in the obtained sequences. The physicochemical properties of the identified family members were analyzed using ExPASy (https://www.expasy.org, accessed on 21 August 2023) [40]. SignalP-4.1 (https://services.healthtech.dtu.dk/services/SignalP-4.1/, accessed on 21 August 2023) [41] and WoLF PSORT (https://wolfpsort.hgc.jp/, accessed on 21 August 2023) were utilized for in silico subcellular localization prediction and to assess the presence of signal peptides [42].

### 4.3. Phylogenetic Analysis of AAAP Genes

First, the *AAAP* family gene IDs in *Oryza sativa* were obtained from the literature [4]. Subsequently, the genome data were acquired from the Ensembl Plants database (https://plants.ensembl.org/Oryza_sativa/Info/Index, accessed on 21 August 2023) and both the CDS and amino acid sequences for the relevant genes were extracted [43]. For phylogenetic tree construction, the AAAP amino acid sequences were imported from *Arabidopsis*, *O. sativa*, and *Vitis vinifera* into MEGA 11. Sequence alignment was conducted using the MUSCLE algorithm, and after determining the optimal model, the neighbor-joining method was employed to construct the phylogenetic tree, setting the number of bootstrap replications to 1000 [44]. Lastly, Evolview (http://www.evolgenius.info/evolview/#login, accessed on 3 September 2023) was utilized to enhance the visualization of the evolutionary tree.

### 4.4. Structure, Conserved Motifs, Sequence Alignment, and Phylogenetic Tree Construction of VvAAAP Family Genes

The VvAAAP amino acid sequences underwent sequence alignment using MUSCLE in MEGA11, followed by optimal model determination. A phylogenetic tree was constructed using the neighbor-joining method, with 1000 bootstrap replications for statistical support [44]. Conserved motifs within the grape VvAAAP proteins were identified using the MEME Suite (https://meme-suite.org/meme/tools/meme, accessed on 21 August 2023), setting the identification of 20 motifs per sequence [45]. TBtools v2.096 software was utilized for the visual mapping of the motifs. The NCBI database (https://www.ncbi.nlm.nih.gov/Structure/bwrpsb/bwrpsb.cgi, accessed on 21 August 2023) was employed to analyze the conserved domains of the family members. Additionally, TBtools was used to illustrate the intron and exon structure of the genes based on the grape GFF3 annotation file.

### 4.5. Chromosome Location, Collinearity, and Duplication Analysis of VvAAAP Family Genes

Utilizing the location information of *VvAAAP* extracted from the grape genome, chromosome mapping was conducted using TBtools v2.096 Gene Location Visualize [46]. The collinear relationships between *Vitis vinifera* and *Arabidopsis*, as well as between *Vitis vinifera* and *O. sativa*, were analyzed using the MCScanX plug-in in TBtools [47].

### 4.6. Analysis and Visualization of Cis-Acting Elements

The 2000 bp sequence upstream of the start codon of *VvAAAPs* was extracted from the grape genome to serve as the promoter sequence. This sequence was subsequently analyzed using PlantCARE (http://bioinformatics.psb.ugent.be/webtools/plantcare/html, accessed on 1 November 2023) for the prediction of *cis*-regulatory elements within the promoter region [48]. Visualization of the components was performed using R 4.1.0.

### 4.7. Analysis of Expression Patterns of VvAAAP Family Genes in Wine Grapes During Development Stages and Under Abiotic Stress

Expression levels of *VvAAAP* family genes in Cabernet Sauvignon, Pinot Noir, and Chardonnay berries were analyzed using transcriptomic data. Each grape variety was evaluated at three berry development stages: EL-33, EL-35, and EL-37. Samples were ground in liquid nitrogen, and total RNA was extracted using the RNAprep Pure Plant Plus Kit (Polysaccharides & Polyphenolics-rich) (TIANGEN, Beijing, China). The concentration, purity, and integrity of the extracted RNA were verified. Transcriptome libraries were constructed using RNA samples and sequenced on a high-throughput Illumina platform to obtain extensive transcriptome sequence data. The sequencing data were preprocessed and aligned to the grape genome to obtain gene expression information. Gene expression levels were quantified as fragments per kilobase of transcript per million mapped fragments (FPKM). The resulting FPKM values were log2 transformed and visualized as heatmaps using the TBtools software heatmap plug-in.

The expression data of *VvAAAP* genes under drought and salt stress were obtained from NCBI (GSE36128 and GSE31677), with Cabernet Sauvignon as the experimental material. For the drought treatment, watering was discontinued at the experiment’s onset and monitored thereafter. The salt stress treatment followed a progressive protocol: initially, plants were watered with 10 mM NaCl and 1 mM CaCl_2_ on day one. From days 2 to 6, the concentration was increased to 20 mM NaCl and 2 mM CaCl_2_ daily. On days 7 and 8, the concentration was further elevated to 55 mM NaCl and 5.5 mM CaCl_2_ daily. From days 9 to 12, plants received 75 mM NaCl and 7.5 mM CaCl_2_ daily. On days 13 and 14, the treatment consisted of 175 mM NaCl and 17.5 mM CaCl_2_, followed by 250 mM NaCl and 25 mM CaCl2 on days 15 and 16. For all treatments, grape bud tips were harvested at 0, 4, 8, 12, and 16 days post-treatment. Total RNA was extracted using the Tris-LiCl method. RNA labeling followed the Affymetrix protocol (http://www.affymetrix.com/support/technical/index.affx, accessed on 20 November 2024). The cDNA synthesis utilized a 3′ One-Cycle cDNA Synthesis kit (Affymetrix p/n 900431) (Affymetrix, Santa Clara, CA, USA), and biotin-labeled cRNA was synthesized using the MEGAscript^®^ IVT Labeling kit (Affymetrix, Santa Clara, CA, USA). Following cRNA fragmentation, hybridization was conducted at 45 °C for 16 h. Chips were cleaned using GeneChip^®^ Fluidics Station 450 (Affymetrix, Santa Clara, CA, USA) and scanned using GeneChip^®^ 3000 Scanner (Affymetrix, Santa Clara, CA, USA). The probe consensus sequence was queried for nucleotide sequences through the Tera-BLASTP program (TimeLogic, Carlsbad, CA, USA)), with probe annotations updated to match the UniProt/TrEMBL protein database. Data from all GeneChip^®^ oligonucleotide arrays were processed using the BioConductor R 1.0.0 package *affy* and the RMA method. Differential gene expression under various stress conditions and control conditions was determined by ANOVA analysis [49].

### 4.8. RT-qPCR Analysis of Certain VvAAAP Family Genes During Developmental Processes and Across Various Tissues

Samples of wine grape varieties, including Cabernet Sauvignon, Pinot Noir, Merlot, Chardonnay, and Muscat Blanc, were collected at various developmental stages and from different tissues. RNA was extracted from the samples according to the method described in Section 4.7. Following quality inspection, the RNA was reverse-transcribed into cDNA using the HiScript III 1st Strand cDNA Synthesis Kit (+gDNA wiper) (Vazyme, Nanjing, China). Subsequently, RT-qPCR-based expression analysis was performed on a Jena qTower3 platform (Analytik Jena AG, Jena, Germany) using ChamQ Universal SYBR qPCR Master Mix (Vazyme, Nanjing, China). The qPCR reaction was conducted in a 20 µL volume with the following program: 95 °C for 30 s, followed by 40 cycles of 95 °C for 5 s and 58 °C for 34 s. The 2^−∆∆Ct^ method was employed to calculate the relative gene expression levels.

## 5. Conclusions

This investigation comprehensively identifies and examines the grape *VvAAAP* family genes, elucidating their structural, evolutionary, expression, and functional diversity. The study identified 60 *AAAP* family genes within the grape genome, distributed across 14 chromosomes and classified into eight subfamilies. Analysis of the promoter sequences reveals that *cis*-elements associated with abiotic and biotic stresses predominantly characterize the promoter regions of most genes. The majority of *VvAAAP* genes demonstrate varied expression patterns across different tissues and developmental stages, indicating their important roles in growth, development, and stress responses. These findings establish a critical foundation for future functional research and applications. Further detailed investigation of the specific mechanisms underlying *VvAAAP* family genes in grape growth, development, and stress responses may provide novel insights and approaches for the genetic enhancement and molecular breeding of grapes.

## Figures and Tables

**Table 1:** The general information and sequence characteristics of 60 *VvAAAP* genes.

8	ID	Protein				Exon	Intron	Instability Index	Aliphatic Index	In-Silico Subcellular Localization	Signal Peptides
		Length (aa)	Mw (D)	pI	GRAVY						
*VvAAAP1*	Vitis01g01152.t01	403	44,078.59	9.18	0.803	3	2	32.04	121.94	Plasma Membrane	No
*VvAAAP2*	Vitis01g01656.t01	338	38,337.29	9.38	0.504	7	6	30.74	96.69	Plasma Membrane	No
*VvAAAP3*	Vitis01g01657.t01	371	41,651.5	9.38	0.595	7	6	35.91	108.33	Vacuole Membrane	No
*VvAAAP4*	Vitis01g01669.t01	360	40,344.17	9.4	0.656	6	5	35.06	106.97	Vacuole Membrane	No
*VvAAAP5*	Vitis01g01676.t01	359	40,166.51	9.21	0.607	7	6	32.93	102.7	Vacuole Membrane	No
*VvAAAP6*	Vitis02g00118.t01	455	49,997.08	7.65	0.629	7	6	40.23	112.11	Plasma Membrane	No
*VvAAAP7*	Vitis03g00189.t01	489	55,151.45	8.94	0.398	8	7	33.88	95.91	Plasma Membrane	No
*VvAAAP8*	Vitis03g00205.t01	512	56,630.07	8.9	0.392	8	7	39.18	100.76	Vacuole Membrane	No
*VvAAAP9*	Vitis03g00324.t01	562	61,306.05	9.48	0.348	5	4	38.89	98.4	Plasma Membrane	No
*VvAAAP10*	Vitis03g00325.t01	493	53,834.7	9.17	0.495	5	4	39.02	105.64	Plasma Membrane	No
*VvAAAP11*	Vitis03g00359.t01	426	46,335.15	6.59	0.842	1	0	32.06	116.34	Vacuole Membrane	No
*VvAAAP12*	Vitis04g00019.t01	483	52,885.9	8.73	0.411	8	7	44.55	101.76	Vacuole Membrane	No
*VvAAAP13*	Vitis04g00020.t01	476	52,469.63	8.75	0.422	8	7	43.97	102.25	Vacuole Membrane	No
*VvAAAP14*	Vitis04g00021.t01	478	52,454.43	8.91	0.486	8	7	42.32	103.18	Vacuole Membrane	No
*VvAAAP15*	Vitis04g00022.t01	410	45,510.37	8.87	0.391	7	6	41.32	95.68	Plasma Membrane	No
*VvAAAP16*	Vitis04g00024.t01	480	52,650.87	8.82	0.501	8	7	41.49	104.25	Vacuole Membrane	No
*VvAAAP17*	Vitis04g01717.t01	526	57,842.88	9.12	0.382	5	4	39.31	99.96	Plasma Membrane	No
*VvAAAP18*	Vitis05g01647.t01	425	45,966.23	6.95	0.691	3	2	29.46	107.55	Plasma Membrane	No
*VvAAAP19*	Vitis05g01648.t01	470	50,974.36	9.2	0.554	3	2	39	113.91	Plasma Membrane	No
*VvAAAP20*	Vitis06g01165.t01	421	46,041.45	6.58	0.773	3	2	42.83	121.33	Plasma Membrane	No
*VvAAAP21*	Vitis06g01608.t01	457	49,448.92	8.68	0.592	7	6	29.93	108.82	Plasma Membrane	No
*VvAAAP22*	Vitis07g00023.t01	451	49,785.13	9.57	0.464	7	6	31.56	101.29	Plasma Membrane	No
*VvAAAP23*	Vitis07g02341.t01	422	45,679.11	7.55	0.774	1	0	33.16	113.15	Plasma Membrane	No
*VvAAAP24*	Vitis07g02360.t01	489	53,832.08	9.02	0.481	7	6	33.62	100.92	Vacuole Membrane	No
*VvAAAP25*	Vitis07g02372.t01	517	56,945.05	8.9	0.468	6	5	35.74	102.4	Vacuole Membrane	No
*VvAAAP26*	Vitis08g01280.t01	478	54,001.95	8.58	0.378	9	8	35	96.13	Plasma Membrane	No
*VvAAAP27*	Vitis08g01632.t01	537	58,572	5.42	0.496	12	11	36.8	112.33	Vacuole Membrane	No
*VvAAAP28*	Vitis09g00857.t01	494	53,498.85	5.85	0.66	1	0	30.58	125.4	Vacuole Membrane	No
*VvAAAP29*	Vitis09g01083.t01	422	45,808.81	8.57	0.916	5	4	35.91	128.65	Plasma Membrane	No
*VvAAAP30*	Vitis10g01757.t01	329	36,117.8	9.48	0.624	5	4	33.97	106.99	Vacuole Membrane	No
*VvAAAP31*	Vitis13g00031.t01	471	53,266.2	8.44	0.413	9	8	31.35	98.75	Plasma Membrane	No
*VvAAAP32*	Vitis13g00162.t01	443	47,802.73	9.19	0.87	5	4	31.93	126.79	Vacuole Membrane	No
*VvAAAP33*	Vitis13g00520.t01	471	51,368.02	7.11	0.509	7	6	32.05	105.9	Plasma Membrane	No
*VvAAAP34*	Vitis13g00680.t01	551	59,856.23	6.11	0.379	14	13	57.01	109.49	Vacuole Membrane	No
*VvAAAP35*	Vitis13g00838.t01	449	48,922.83	9.22	0.693	7	6	25.6	117.26	Plasma Membrane	No
*VvAAAP36*	Vitis13g00839.t01	450	49,085.86	9.03	0.662	7	6	25.85	114.64	Plasma Membrane	No
*VvAAAP37*	Vitis13g00841.t01	449	48,832.71	8.96	0.685	7	6	26.18	115.32	Plasma Membrane	No
*VvAAAP38*	Vitis14g01612.t01	462	50,204.08	7.09	0.661	6	5	34.07	115.84	Vacuole Membrane	No
*VvAAAP39*	Vitis14g02003.t01	357	39,563.65	8.8	0.594	8	7	34.45	103.28	Vacuole Membrane	No
*VvAAAP40*	Vitis14g02396.t01	428	46,939.67	6.21	0.827	3	2	33.9	120	Plasma Membrane	No
*VvAAAP41*	Vitis14g02397.t01	428	47,075.55	7.02	0.661	3	2	38.74	120	Plasma Membrane	No
*VvAAAP42*	Vitis14g02399.t01	556	61,064.09	6.98	0.554	8	7	41.92	119.24	Plasma Membrane	No
*VvAAAP43*	Vitis14g02399.t02	474	52,318.02	7.51	0.585	7	6	41.05	121.77	Vacuole Membrane	No
*VvAAAP44*	Vitis18g00135.t01	526	58,040.68	8.87	0.519	5	4	35.84	106.94	Plasma Membrane	No
*VvAAAP45*	Vitis18g00137.t01	515	56,432.66	9.08	0.544	4	3	39.32	108.66	Chloroplast	No
*VvAAAP46*	Vitis18g00255.t01	483	53,439.93	9.15	0.474	7	6	38.53	102.22	Plasma Membrane	No
*VvAAAP47*	Vitis18g00349.t01	464	52,574.37	9	0.412	8	7	29.13	95.8	Plasma Membrane	No
*VvAAAP48*	Vitis18g00673.t01	483	53,480.38	8.82	0.406	7	6	36.04	98.72	Vacuole Membrane	No
*VvAAAP49*	Vitis18g00695.t01	449	48,931.63	7.11	0.727	5	4	33.46	118.98	Vacuole Membrane	No
*VvAAAP50*	Vitis18g00959.t01	514	56,829.77	9.5	0.498	5	4	36.16	100	Plasma Membrane	No
*VvAAAP51*	Vitis18g02524.t01	455	50,327.13	9.19	0.596	6	5	33.44	109.25	Vacuole Membrane	No
*VvAAAP52*	Vitis18g02525.t01	454	50,973.1	9.4	0.575	6	5	36.2	106.06	Vacuole Membrane	No
*VvAAAP53*	Vitis18g02527.t01	459	50,992.89	9.19	0.475	6	5	34.16	101.15	Plasma Membrane	No
*VvAAAP54*	Vitis19g00923.t01	811	89,337	7.69	0.438	15	14	35.72	96.09	Vacuole Membrane	No
*VvAAAP55*	Vitis19g00923.t02	613	67,686.92	7.44	0.473	11	10	35.24	96.39	Vacuole Membrane	No
*VvAAAP56*	Vitis19g01667.t01	404	44,117.63	5.77	0.891	3	2	30.37	123.76	Vacuole Membrane	No
*VvAAAP57*	Vitis19g01668.t01	407	44,792.07	8.37	0.804	4	3	32.86	123.59	Plasma Membrane	No
*VvAAAP58*	Vitis19g01670.t01	582	64,027.08	8.61	0.829	6	5	33.11	125.81	Plasma Membrane	No
*VvAAAP59*	Vitis19g01670.t02	532	58,720.98	8.53	0.861	5	4	32.61	128.08	Vacuole Membrane	No
*VvAAAP60*	Vitis19g01671.t01	406	44,503.01	8.29	0.844	5	4	30.88	125.05	Plasma Membrane	No

Length: amino acid length; MW: molecular weight; pI: isoelectric point; GRAVY: grand average of hydropathicity.

## Data Availability

Data are contained within the article.
